# Effect of noradrenaline on propofol-induced mitochondrial dysfunction in human skeletal muscle cells

**DOI:** 10.1186/s40635-022-00474-3

**Published:** 2022-11-08

**Authors:** Adéla Krajčová, Christine Skagen, Valér Džupa, Tomáš Urban, Arild C. Rustan, Kateřina Jiroutková, Bohumil Bakalář, G. Hege Thoresen, František Duška

**Affiliations:** 1 Department of Anaesthesia and Intensive Care of the Third Faculty of Medicine and Královské Vinohrady University Hospital, OXYLAB-Laboratory for Mitochondrial Physiology, Charles University, Prague, Czech Republic; 2 Section for Pharmacology and Pharmaceutical Biosciences, Department of Pharmacy, University of Oslo, Oslo, Norway; 3 Department of Orthopaedics and Traumatology of The Third Faculty of Medicine and Královské Vinohrady University Hospital, Charles University, Prague, Czech Republic; 4 Department of Pharmacology, Institute of Clinical Medicine, University of Oslo, Oslo, Norway

**Keywords:** Propofol infusion syndrome, Noradrenaline, Mitochondrial dysfunction, Skeletal muscle, Critical illness

## Abstract

**Background:**

Mitochondrial dysfunction is a hallmark of both critical illness and propofol infusion syndrome and its severity seems to be proportional to the doses of noradrenaline, which patients are receiving. We comprehensively studied the effects of noradrenaline on cellular bioenergetics and mitochondrial biology in human skeletal muscle cells with and without propofol-induced mitochondrial dysfunction.

**Methods:**

Human skeletal muscle cells were isolated from vastus lateralis biopsies from patients undergoing elective hip replacement surgery (*n* = 14) or healthy volunteers (*n* = 4). After long-term (96 h) exposure to propofol (10 µg/mL), noradrenaline (100 µM), or both, energy metabolism was assessed by extracellular flux analysis and substrate oxidation assays using [^14^C] palmitic and [^14^C(U)] lactic acid. Mitochondrial membrane potential, morphology and reactive oxygen species production were analysed by confocal laser scanning microscopy. Mitochondrial mass was assessed both spectrophotometrically and by confocal laser scanning microscopy.

**Results:**

Propofol moderately reduced mitochondrial mass and induced bioenergetic dysfunction, such as a reduction of maximum electron transfer chain capacity, ATP synthesis and profound inhibition of exogenous fatty acid oxidation. Noradrenaline exposure increased mitochondrial network size and turnover in both propofol treated and untreated cells as apparent from increased co-localization with lysosomes. After adjustment to mitochondrial mass, noradrenaline did not affect mitochondrial functional parameters in naïve cells, but it significantly reduced the degree of mitochondrial dysfunction induced by propofol co-exposure. The fatty acid oxidation capacity was restored almost completely by noradrenaline co-exposure, most likely due to restoration of the capacity to transfer long-chain fatty acid to mitochondria. Both propofol and noradrenaline reduced mitochondrial membrane potential and increased reactive oxygen species production, but their effects were not additive.

**Conclusions:**

Noradrenaline prevents rather than aggravates propofol-induced impairment of mitochondrial functions in human skeletal muscle cells. Its effects on bioenergetic dysfunctions of other origins, such as sepsis, remain to be demonstrated.

**Supplementary Information:**

The online version contains supplementary material available at 10.1186/s40635-022-00474-3.

## Introduction

Mitochondrial dysfunction in skeletal muscle is a hallmark of sepsis [1], ICU-acquired skeletal muscle dysfunction, acute lung injury, acute renal failure, and critical illness-related immune function dysregulation [2]. An association has been found 20 years ago [1] between the degree of mitochondrial dysfunction and the dose of noradrenaline that the patients were receiving.

The effects of drugs frequently used in ICU on cellular bioenergetics are understudied, but propofol is known to induce mitochondrial dysfunction [3–8], which, in its extreme form may result in propofol infusion syndrome (PRIS), a rare but potentially lethal complication [9–11]. Typical features of the syndrome include metabolic acidosis, arrhythmias, Brugada-like pattern on electrocardiogram, hypertriglyceridemia, fever, rhabdomyolysis, hepatomegaly, cardiac and/or renal failure [9–11]. The risk of the syndrome increases with rising dose and duration of propofol administration, low carbohydrate intake, inborn mitochondrial diseases, critical illness and concomitant treatment with corticosteroids [9, 12, 13]. In addition, most patients with PRIS were simultaneously treated with high doses of intravenous catecholamines leading to concern that they might be one of the triggering factors of the syndrome and could be associated with mortality [9, 12]. It remains unclear whether high doses of noradrenaline could have causally contributed to the development of PRIS or whether it is an epiphenomenon.

Noradrenaline is the first-choice vasopressor in critically ill patients [14–16] and propofol is used ubiquitously in ICUs. Therefore, in this study we sought out to investigate the effects on cellular bioenergetics of 4 days of exposure to pharmacological concentrations of propofol and noradrenaline.

## Materials and methods

### Study subjects

Skeletal muscle tissue biopsies were obtained from patients undergoing elective hip replacement surgery at the Department of Orthopaedic Surgery of Královské Vinohrady University Hospital in Prague (*n* = 14). Specimens were taken by open technique from *vastus lateralis* muscle (sample ~̴ 300 mg) during surgery. In addition, for [1-^14^C]palmitic acid and [^14^C(U)]lactic acid experiments, samples from *vastus lateralis* muscle were obtained under local anaesthesia by Bergström technique (*n* = 4) at Norwegian School of Sport Sciences, Oslo, in cooperation with Department of Pharmacy, University of Oslo. The study protocols were approved by respective REBs in both institutions. All subjects provided a prospective written informed consent. Detailed characteristics of skeletal muscle donors are described in Additional file 1: see Table S1.

### Isolation and cultivation of human skeletal muscle cells

Skeletal muscle cells were isolated and cultured on gelatin-coated flasks or collagen-coated 24-well tissue culture plates as previously described [17] (for details see Additional file 1). Upon 80–90% confluency, differentiation of cells was induced by reducing serum concentration in cell culture medium [17]. Differentiated multinucleated myotubes were then treated with either 10 µg/mL propofol, 0.1 mM noradrenaline (NA) or both agents for 96 h. The cells then underwent several experimental procedures, as described below. For the confocal laser scanning microscopy experiments, we used proliferating myoblasts. All experiments were performed within 5 passages after isolation of cells. All chemicals were purchased from Merck Millipore (Darmstadt, Germany) or Life Technologies (Gaithersburg, MD, US), unless otherwise stated.

Viability assays and drug preparation is described in the Additional file 1. Initially, we tested a range of propofol concentrations, from those resembling propofol levels in human plasma during sedation and anaesthesia (2.5 and 10 µg/mL) [18, 19] to supratherapeutic concentrations. Given that high propofol concentrations (10 ˃ µg/mL) impair cell viability and there is no significant difference between the therapeutic concentrations (Additional file 1: see Figs. S1, S2), we used only 10 µg/mL for further experiments. Similarly, we studied the effect of a range of noradrenaline concentrations (0.5; 1; 10 and 100 µM). Noradrenaline had no significant impact on cell survival in a dose-dependent manner (Additional file 1: see Fig. S3) and hence we used 100 µM noradrenaline for further experiments.

### Cellular bioenergetics

**Fig. 1:**
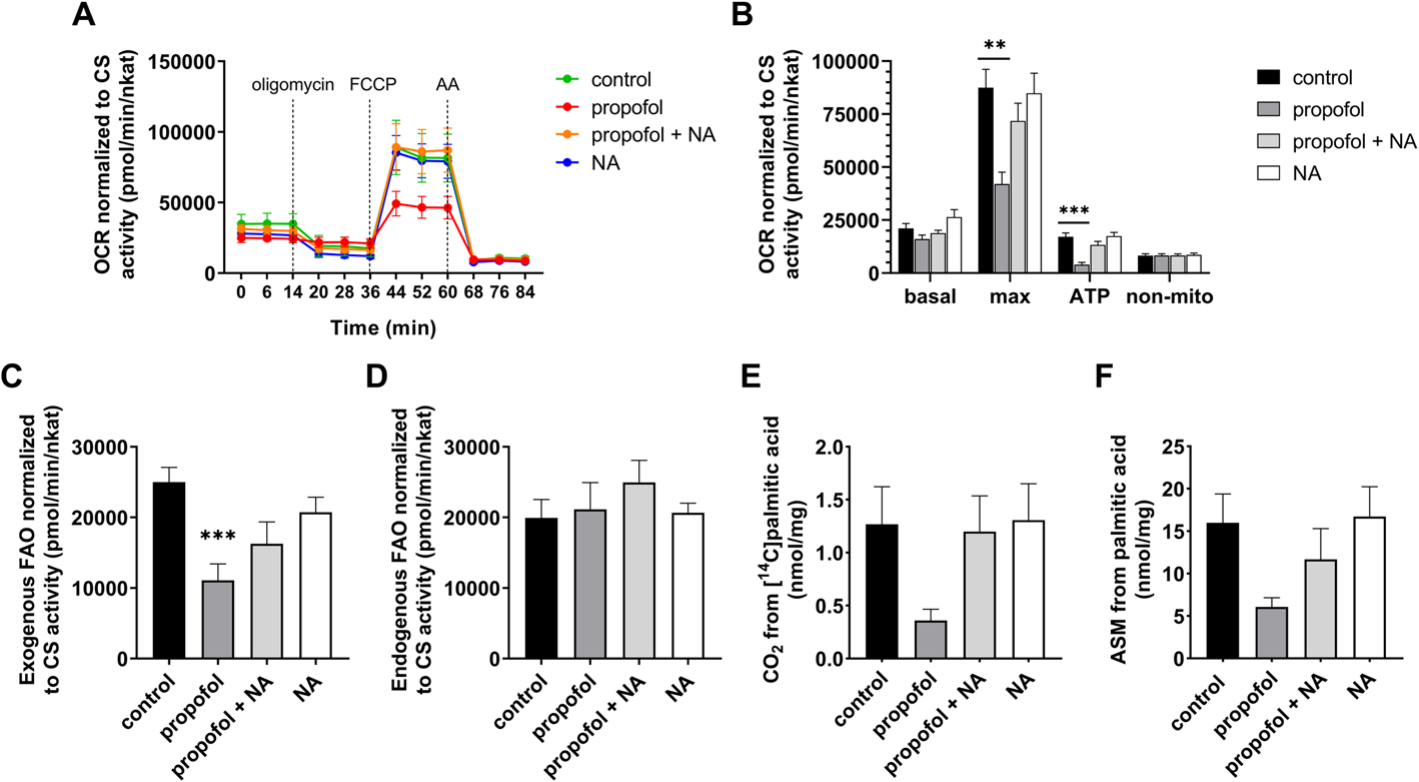
Extracellular flux analysis and substrate oxidation assays. **A** Real-time measurement of OCR at baseline and after sequential injection of oligomycin, FCCP and antimycin A. Each data-point represents the mean of 7 subjects measured in tri- or tetraplicates. Values are normalized to mitochondrial content (CS activity). **B** Global mitochondrial parameters. Basal respiration, maximal respiratory capacity, ATP production and non-mitochondrial respiration determined from OCRs shown in part A. **C** Exogenous oxidation of fatty acids after palmitate addition to the medium during measurement. **D** Endogenous oxidation of fatty acids in palmitate-free medium. **E** CO_2_ production from [^14^C]palmitic acid (complete oxidation). **F** Oxidation of [^14^C]palmitic acid to ASM (incomplete oxidation). Error bars in each graph indicate standard error of the mean. **p < 0.01, *** *p* < 0.001 vs. control group. *AA* antimycin A, *FCCP*  carbonyl cyanide-4- (trifluoromethoxy)phenylhydrazone, *FAO*  fatty acid oxidation, *NA*  noradrenaline, *OCR*  oxygen consumption rate

We used XF-24 Extracellular Flux Analyzer (Agilent Technologies Inc., Santa Clara, CA, US) to measure oxygen consumption rate (OCR) in living cells seeded on 24-well plate at 37 °C [20–22] at baseline and after a sequential addition of up to four compounds [20–22]. We performed three types of assays (measurements in tri- or tetraplicates from 7 subjects for each protocol): Global mitochondrial parameters, i.e. basal respiration, ATP production, maximal respiratory capacity and non-mitochondrial respiration, were determined by sequential injection of F_O_F_1_ ATPase inhibitor oligomycin [1 µM], an uncoupler of the respiratory chain carbonyl cyanide-4-(trifluoromethoxy)phenylhydrazone (FCCP; [1 µM]) and complex IV inhibitor antimycin A [4 µM] (see Fig. 1, part A and B; for detailed information see Additional file 1). Exogenous fatty acid oxidation (FAO) was determined in carnitine-supplemented medium by the addition of an uncoupler FCCP [1 µM] followed by a stepwise addition of sodium palmitate (to a final concentration of 200 µM; see Fig. 1, part C) and FAO inhibitor etomoxir [40 µM]. Endogenous fatty acid oxidation was determined in palmitate-free medium by sequential addition of FCCP [1 µM] and etomoxir [40 µM] (see Fig. 1, part D). In both protocols, FAOs were calculated as the decrement of OCR after the addition of etomoxir. Where appropriate, OCR was normalized to citrate synthase (CS) activity determined spectrophotometrically (CS Assay kit, Merck Millipore, Darmstadt, Germany) [23] in cell pellets.

### Substrate oxidation assays and acid-soluble metabolites

Fully differentiated myotubes were incubated with either 100 µM [^14^C(U)]lactic acid (0.5 µCi/ml) or 100 µM [1-^14^C]palmitic acid (0.5 µCi/ml) in DMEM-GlutaMAX™ (low glucose) containing 10 mM HEPES, 10 µM BSA and 1 mM L-carnitine for 4 h. Palmitic acid was bound to BSA at a ratio of 2.5:1. Following the 4-h incubation period, the cells were lysed and medium containing radiolabelled palmitic acid was collected and saved for oxidation and acid-soluble metabolites (ASM) measurements. For the lactate experiments, cell-associated radioactivity plus CO_2_ production reflects total cellular uptake of [^14^C(U)]lactic acid, while cell-associated radioactivity plus CO_2_ plus ASM reflects total cellular uptake of [^14^C]palmitic acid.

#### Analysis of cell-associated radioactivity and CO_2_ formation

100 µl medium containing radioactive substrates was transferred to a multi-well plate, sealed and frozen at − 20 °C following the 4 h incubation. CO_2_ production was measured by adding 40 µl of 1 M perchloric acid (HClO_4_) to the frozen medium, where the CO_2_ was captured by a 96-well UniFilter-96 GF/B microplate that was mounted on top of the multi-well plate [24]. The mixture was incubated at room temperature for 3 h to trap radiolabelled CO_2_. The CO_2_ produced during the 4 h of incubation with [1-^14^C]palmitic and [^14^C(U)]lactic acid was captured by the sodium bicarbonate buffer system in the cell medium. After adding HClO_4_ to the frozen medium, CO_2_ was released and captured in the UniFilter-96 GF/B microplate. The cell lysates were used to measure the cell-associated radioactivity. Radioactivity was measured by liquid scintillation (2450 MicroBeta [24] scintillation counter, PerkinElmer).

#### Analysis of acid-soluble metabolites

The remaining radioactive medium containing [^14^C]palmitic acid was used to measure ASM, which mainly consist of tricarboxylic acid cycle metabolites and reflect incomplete FAO. The collected radiolabelled incubation medium (100 µl) was transferred to Eppendorf tubes, added 300 µL HClO_4_ and 30 µL BSA (6%) before being centrifuged. The supernatant was then counted by liquid scintillation (Packard Tri-Carb 1900 TR, PerkinElmer).

### Confocal laser scanning microscopy and live-cell imaging

**Fig. 2:**
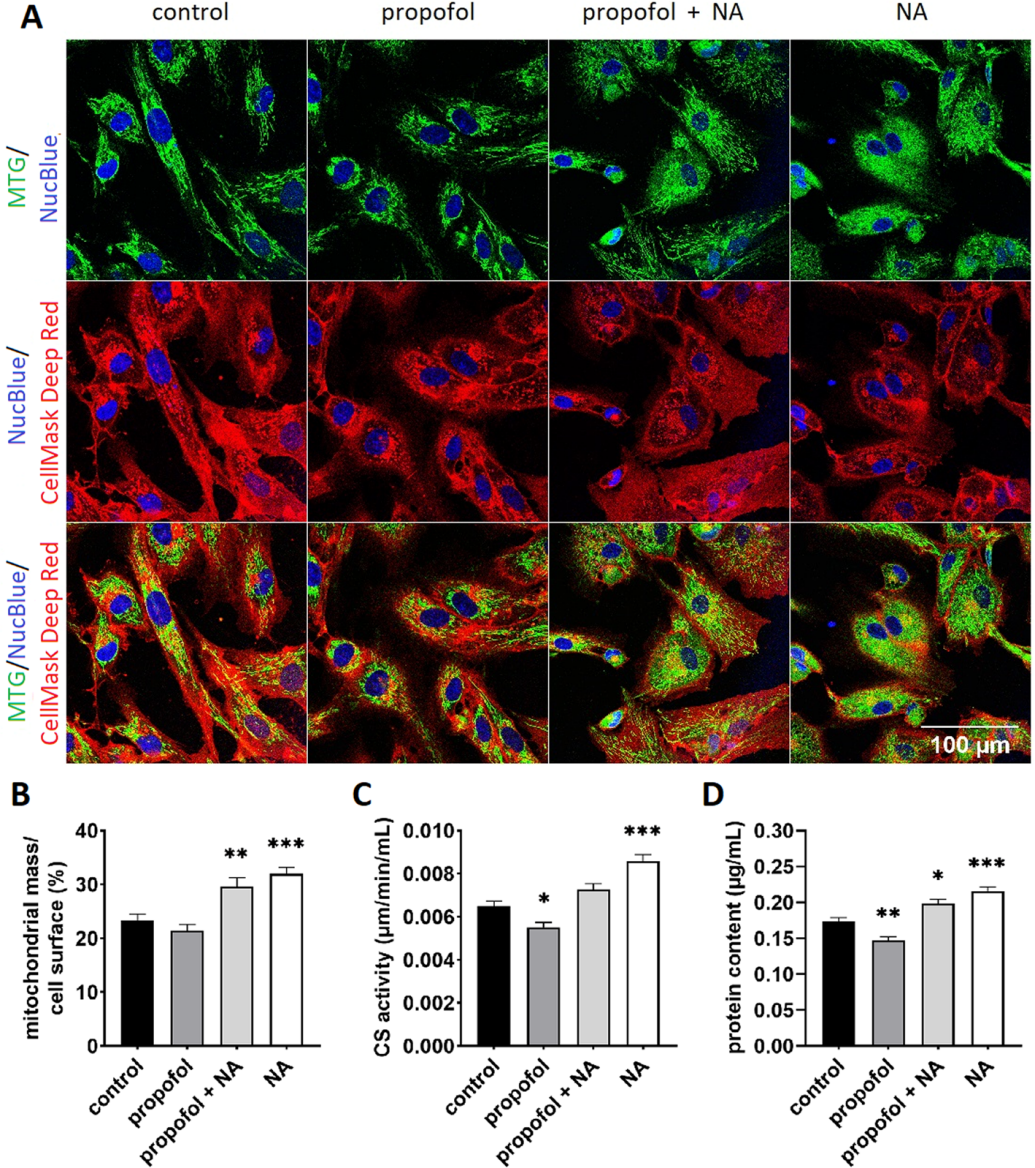
**A** and **B** Analysis of mitochondrial mass by confocal imaging of exposed myoblasts. **A** Representative confocal images of each channel after dual staining with Mitotracker Green™ FM (accumulating in mitochondria) and CellMask™ Deep Red (binding into the cell membrane). Additionally, all cells were stained with nuclear blue-fluorescent probe NucBlue. Experiments were performed at least at 50 cells per each condition from 3 independent measurements (= cells established from 3 individual subjects). **B** Mitochondrial mass calculated as a fraction (%) of a cell surface area in 2D cross-sectional images. Mitochondrial mass (mitochondrial footprint) was analysed as a sum of positive pixels (binary image) per cell representing mitochondrial area using ImageJ™ tool “MINA”. **C** Activity of CS enzyme measured spectrophotometrically. **D** Total protein content from frozen cell pellets was determined using Bradford assay as described elsewhere [53]. For both CS activity and protein content measurement, experiments were performed in *n* = 7 replicates in tri- or tetraplicates. Error bars indicate standard error of the mean. *p < 0.05, **p < 0.01, ***p < 0.001 vs. control group. *CS* citrate synthase, *MTG* MitoTracker Green™ FM, *NA* noradrenaline

Microscopy of cell lines at growth conditions (37 °C and 5% CO_2_) has been performed using a 63 × oil immersion objective (*Leica TCS SP5* system, Leica Microsystems). For determination of mitochondrial mass we used MitoTracker™ Green FM (excitation at 488 nm), reflecting mitochondrial mass regardless of mitochondrial membrane potential [25, 26]. In addition, CellMask™ Deep Red Plasma Membrane Stain (excitation at 650 nm) labelling cellular plasma membrane allowed to determine the proportion of the cytoplasm filled with the mitochondrial network [27] (see Fig. 2, part A and B). Production of reactive oxygen species (ROS) [28–30] was determined by loading cells with a reduced non-fluorescent dye MitoTracker Red CM-H2XRos (excitation at 561; see Fig. 4), that fluoresces upon oxidation [29, 30]. Mitochondrial membrane potential ((∆ψ_m_*)* was determined as a ratio of fluorescence intensity after staining the cells with positively charged red dye tetramethylrhodamine ethyl ester (TMRE; excitation at 549 nm) and MitoTracker™ Green FM ( Additional file 1: see Fig. S6). Co-localization of mitochondria with lysosomes during mitophagy was determined in 2D cross-sectional confocal images using the ImageJ™ tool (see Fig. 5) as the fraction of mitochondria, stained with MitoTracker™ Green FM (excitation at 488 nm), that overlapped with lysosomes, stained with LysoTracker™ Deep Red (excitation at 651 nm). Detailed protocols of confocal microscopy are in the Additional file 1. In addition, MitoTracker ™ Green FM-staining of cells was used to determine mitochondrial volume by flow cytometry (BD FACSVerse flow cytometer, BD Biosciences, CA, USA).

### Statistical analysis

All data sets were tested for normality of distribution. One-way ANOVA with Dunnett’s post hoc test or Kruskal–Wallis with Dunn’s post hoc test were used, as appropriate. Differences at *p* < 0.05 were considered significant. Images from confocal laser scanning microscopy were analysed using Image J (Fiji) software, whilst GraphPad Prism 8.0.1 (GraphPad Software Inc., La Jolla, CA, US) was used for graphs and statistics. Image brightness or contrast were not altered in any quantitative image analysis protocols.

## Results

### Effects on cell viability and bioenergetic profile

The concentrations of propofol (10 µg/mL) and noradrenaline (0.1 mM), which we used in all assays, did not affect the viability of the cells (Additional file 1: see Fig. S1).

#### Respiratory chain function indices

Basal OCR normalized to CS activity was not significantly different among the groups (see Fig. 1A). In line, there were no significant changes of [^14^C]lactic acid oxidation or uptake across the experimental conditions (Additional file 1: see Fig. S4). Propofol exposure led to significant reduction of both ATP production at rest and of the maximum respiratory chain capacity. These effects were diminished by co-exposure to noradrenaline, whilst exposure to noradrenaline alone did not have any effects on mitochondrial functional indices (see Fig. 1B).

#### Fatty acid oxidation

Similar pattern of inhibition by propofol, which is mitigated by co-incubation with noradrenaline, have been observed for oxidation of exogenous palmitate by both extracellular flux analysis and by [^14^C] palmitic acid techniques (see Fig. 1C, E–F). Uptake of [^14^C] palmitic acid was not affected by neither compound (Additional file 1: see Fig. S4) and there was no significant alteration of endogenous FAO, either (see Fig. 1D).

### Effects on mitochondrial mass

Microscopic analysis revealed that compared to control cells, noradrenaline alone or in combination with propofol significantly increased mitochondrial mass to 124 ± 3% and 114 ± 3%, respectively, whilst in propofol-exposed cells mitochondrial mass was 85 ± 4% of that in control cells (see Fig. 2A, B). Measurements of CS activity (see Fig. 2C) and protein content (see Fig. 2D) confirmed these results. There were 132 ± 4% and 112 ± 4% increases compared to control cells in cells treated with noradrenaline alone and noradrenaline plus propofol, respectively, and a reduction to 85 ± 4% in cells treated with propofol alone. In 2D confocal microscopy images, mitochondria occupied 32 ± 1% and 30 ± 2% of the cell surface in noradrenaline- and noradrenaline plus propofol-exposed cells, respectively, compared to 23 ± 2% and 21 ± 1% in propofol treated and control cells, respectively. As flow cytometry with MitoTracker™ Green FM staining suggested that these changes were mainly reflective of alterations in mitochondrial volume (Additional file 1: see Fig. S5), we explored in detail how interactions between propofol and noradrenaline affect mitochondrial morphology and turnover, reflected by mitophagy.

### Effects on mitochondrial morphology

**Fig. 3:**
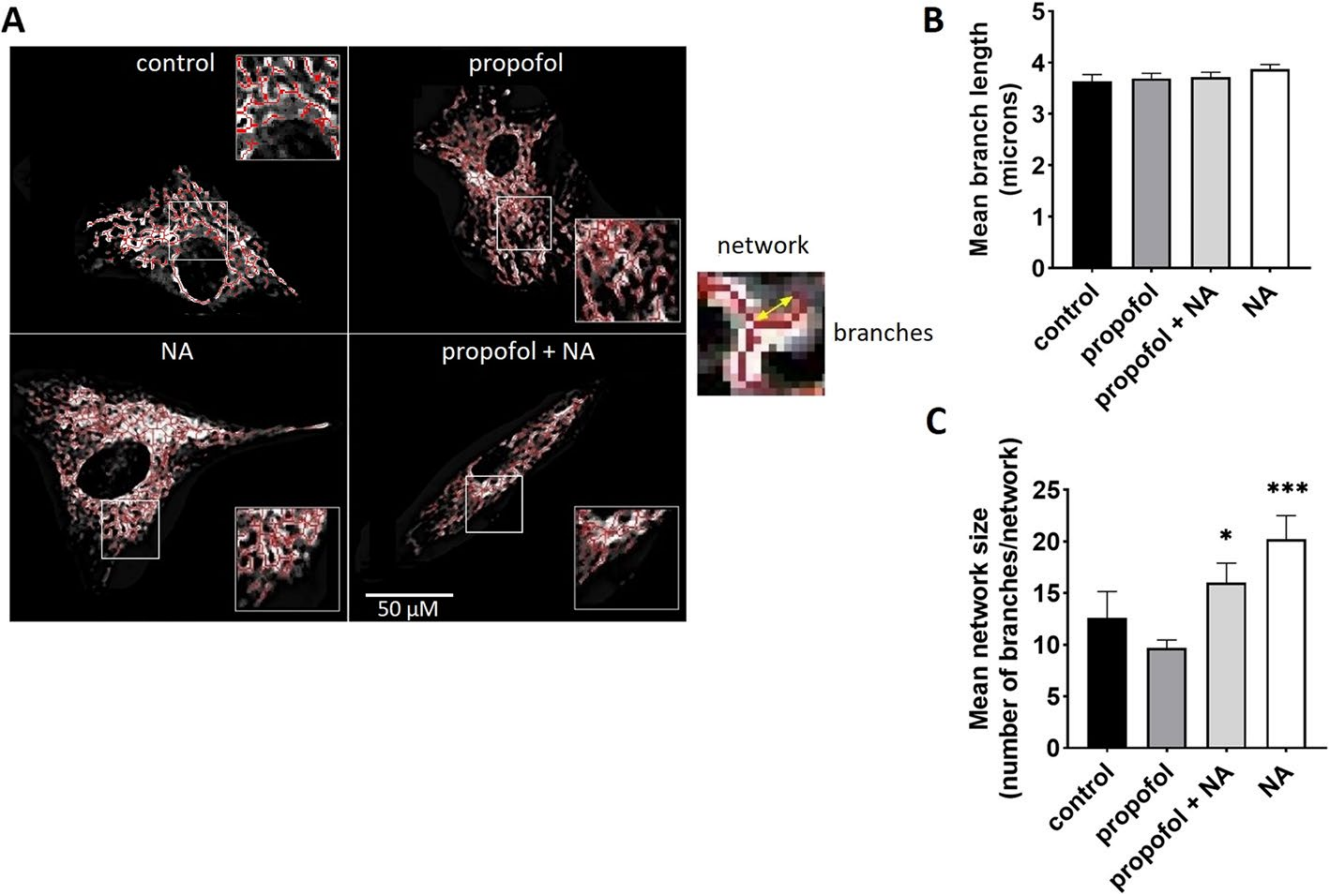
Mitochondrial morphology. Confocal images of myoblasts stained with MitoTracker™ Green FM analysed with the ImageJ™ plugin “MINA”. **A** Representative skeletonized images show mitochondrial network in different groups (left). Measurements were performed at least at 50 cells per each group from 3 independent experiments (= 3 individual subjects). Yellow arrow (right) shows one of the three branches in an example of mitochondrial network. **B** and **C** Mean branch length and mean network size (= number of branches per network) were analysed in each cell separately. Error bars indicate standard error of the mean. *p < 0.05, ***p < 0.001 vs. control group. *NA* noradrenaline

There were no changes of mean branch length across experimental conditions (~ 3.7 ± 0.1 microns for all groups), but the exposure to noradrenaline with or without propofol increased the number of branches per each individual network, and thereby led to an increase in the mean network size (see Fig. 3).

### Effects on mitophagy

**Fig. 4:**
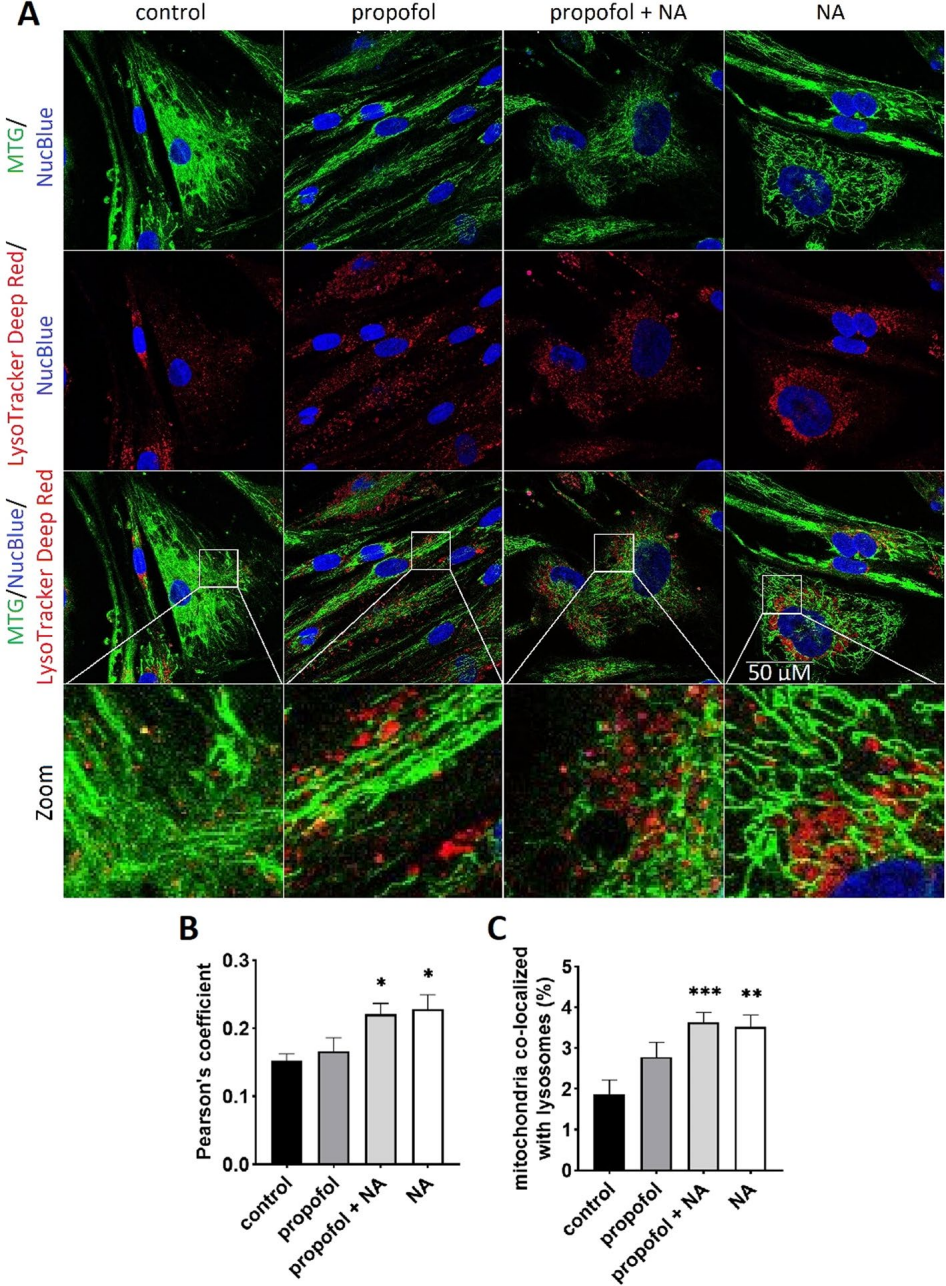
Co-localization of mitochondria with lysosomes. **A** Representative confocal images of each channel after dual staining with Mitotracker Green™ FM (accumulating in mitochondria) and LysoTracker™ Deep Red (binding to acidic lysosomes). Additionally, all cells were stained with blue-fluorescent probe NucBlue to label nuclei. Experiments were performed at least at 40 cells per each condition from 3 independent measurements (= 3 individual subjects). **B** Pearson’s coefficient, characterizing a degree of overlap between labelled mitochondria and lysosomes. The quantification of the co-localization was performed using ImageJ™ plugin “JACoP”. **C** Percent co-localization was calculated by total area of co-localized lysosomes (red channel) over total area of mitochondria (green channel). Error bars indicate standard error of the mean. *p < 0.05, **p < 0.01, ***p < 0.001 vs. control group. *NA* noradrenaline

Since autophagosomes eventually fuse with lysosomes, co-localization analysis of mitochondria with lysosomal markers could be used to monitor mitophagy [31]. Co-localization of mitochondria with lysosomes was significantly higher in noradrenaline and noradrenaline plus propofol groups (3.5 ± 0.3% and 3.6 ± 0.2% for noradrenaline and noradrenaline with propofol, respectively) compared to propofol-exposed (2.8 ± 0.4%) and control cells (1.9 ± 0.4%), respectively. In line, Pearson’s coefficient characterizing a degree of overlap, was higher in noradrenaline-exposed cells (see Fig. 4).

### Effects on reactive oxygen species production and mitochondrial membrane potential (∆ψ_m_)

**Fig. 5:**
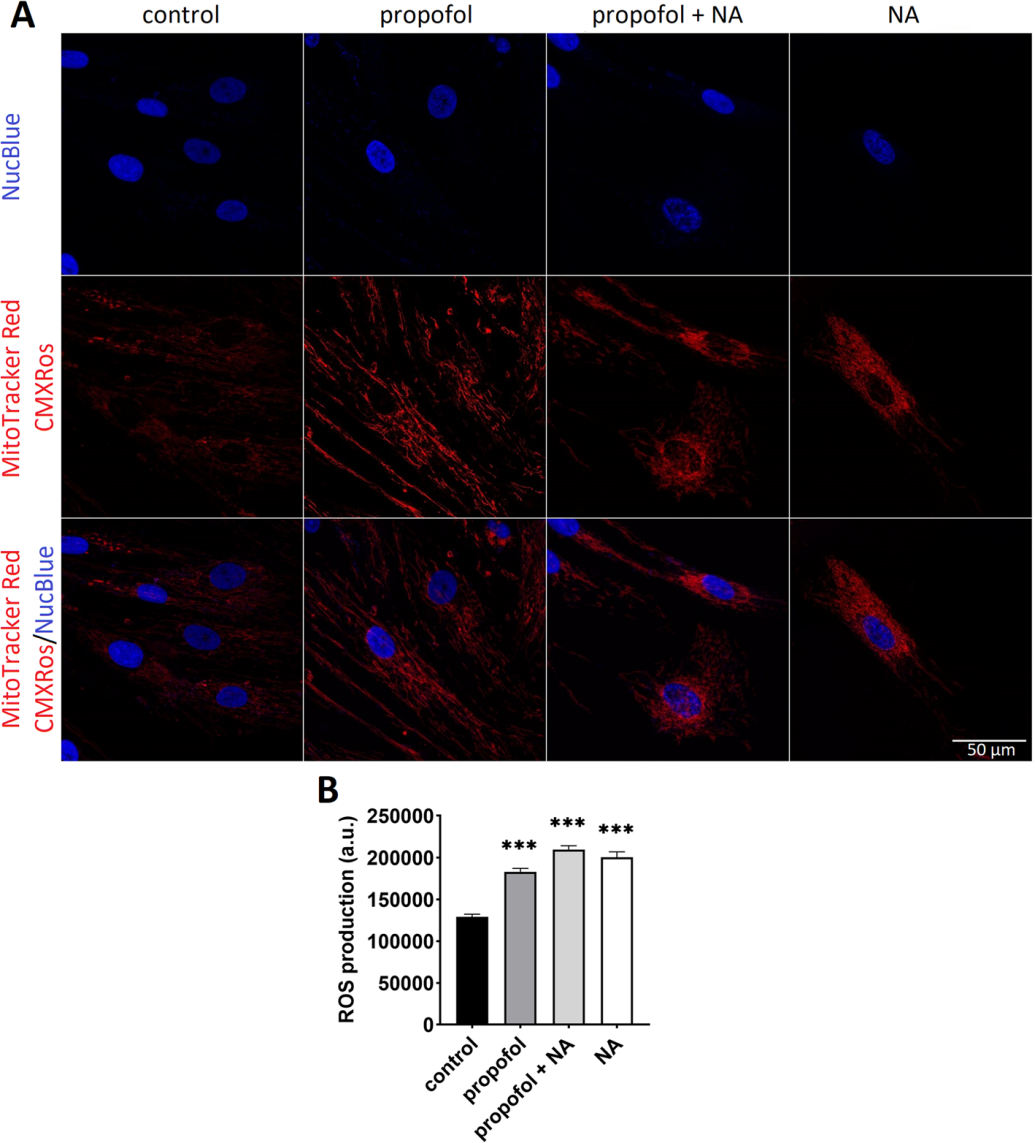
ROS production. **A** Representative confocal images from myoblasts stained with MitoTracker Red CM-H2XRos to detect accumulation of mitochondrial specific-reactive oxygen species. Experiments were performed on at least 90 cells per group from *n* = 3 independent experiments (with cells from 3 individual subjects). **B** Determination of ROS production in different groups. Error bars indicate standard error of the mean. ***p < 0.001 vs. control group. *NA* noradrenaline

Compared to control cells, all experimental exposures led to a significant increase of ROS production (to 142–162% of values in control cells; see Fig. 5) and to a reduction of the mitochondrial membrane potential (Additional file 1: see Fig. S6).

## Discussion

The main finding of this study is that noradrenaline does not potentiate the adverse effects of propofol on cellular bioenergetics—a hypothesis generated by the finding of association between co-exposure to high-dose noradrenaline with mortality of PRIS [9, 13]. Our data support the alternative hypothesis [12] that the need of vasopressor support in patients with fatal PRIS is an epiphenomenon rather than causal contributor to mortality. In fact, we have found a range of effects of noradrenaline on bioenergetics that preserve mitochondrial function under stress (in our experiment induced by exposure of cells to propofol), that can be of importance for the critically ill in general. Not only because noradrenaline and propofol are among the most used drugs in intensive care units, but also because bioenergetic failure has long been known as a hallmark of acute and protracted critical illnesses [1]. Our finding sheds new light into the understanding of the effects of noradrenaline and propofol on a range of aspects of mitochondrial biology.

For our experiments we used a well-established [8] ex vivo model of human skeletal muscle—cultured myotubes differentiated from myoblasts isolated form vastus lateralis biopsies of metabolically healthy patients undergoing hip replacement surgery and healthy volunteers. We exposed those cells for 4 days to both drugs and assessed their impact on mitochondrial mass, morphology, dynamics and function. Not surprisingly and in line with our previous results [8], propofol decreased both ATP production at rest and the maximal respiratory capacity of the electron transport chain, and caused a profound inhibition of the ability to oxidize exogenous palmitate. Noradrenaline significantly counteracted those effects by several ways.

Firstly, noradrenaline increased mitochondrial network mass and size, most likely by increased mitochondrial turnover, as evidenced by increased mitophagy. Several studies have found that catecholamine-induced stimulation of β-adrenergic receptors promotes mitochondrial biogenesis in both skeletal muscle [32, 33] and other tissues [34, 35]. In keeping with this, noradrenaline significantly increased mitochondrial content what we confirmed by three independent techniques. In our study, we also looked at mitochondrial network architecture. Microscopic analysis revealed that in noradrenaline-exposed cells mitochondrial networks exhibited more branches rather than increased elongation compared to control cells. Increased mitochondrial amount could result either from accelerated biogenesis or a slower degradation [31]. We observed an increased co-localization of lysosomes with mitochondria which could be an indicator of a triggering mitophagy [31]. Traditionally, lysosomes have been considered acidic organelles necessary for the autophagy and degradation of cellular components [36]. However, recent studies have shown that lysosomes could serve as the nutrient reservoirs with the potential anabolic effect in skeletal muscle and other tissues [37]. Dynamic formation of inter-organelle membrane contact sites between mitochondria and lysosomes allows a shuttle of amino acids, lipids and ions such as Ca^2+^ between the two organelles [38, 39]. The interaction of mitochondria with lysosomes might therefore play a role in improved metabolic regulation and substrate availability.

On that note, the protective effects of noradrenaline on propofol-exposed cells were still apparent even after adjustment to mitochondrial content. In particular, noradrenaline effectively counteracted propofol inhibitory effect on the oxidation of palmitoyl-carnitine, added to the media. Noradrenaline increases lipolysis in adipose tissue [40, 41], but the results of the studies of effects on intramyocellular lipolysis have been contentious [42–44]. In our study, we did not observe any increase of oxidation of endogenous fatty acids, which would suggest that noradrenaline stimulated intramyocellular lipolysis. This is further supported by the lack of effect of noradrenaline on the size and the number of lipid droplets in human myoblasts (Additional file 1: see Fig. S7). Noradrenaline is known to directly enhance the activity of carnitine palmitoyl transferase 1 (CPT1) [45], an enzyme indispensable for long-chain fatty acids transport to mitochondria, which seems to be inhibited by propofol [46–48]. Our data are consistent with noradrenaline preserving the exogenous FAO in propofol-exposed cells by preserving and activating transport of long-chain fatty acid transport to mitochondria by CPT1.

The main limitation of our study is that we only used a model of a single organ derived from biopsies of subjects that were not critically ill. This indeed severely limits the generalizability of our results. Moreover, ex vivo conditions may be far from representative of in vivo physiology. Yet, unlike other adverse effects of drugs, we believe that bioenergetic effects are important, relevant, and understudied and our study brings important data into the field. Further studies should focus of interactions between drugs frequently used in ICUs and mitochondrial dysfunction induced by critical illness itself. Also, it must be noticed that the concentrations used for noradrenaline exposure in our experiments exceeded therapeutic plasma levels during noradrenaline infusion in critically ills (up to 300 nM) [49–51]. However, noradrenaline concentration in the synaptic clefts in skeletal muscle can be much higher [52].

In conclusion, we for the first time investigated the effects on mitochondrial biology of long-term exposure of human skeletal muscle cells to propofol and noradrenaline. We have shown that noradrenaline does not worsen propofol-induced cellular dysfunction, but in fact, it is able to counteract most adverse effects of propofol on cellular bioenergetics by increasing the mitochondrial turnover and mitochondrial mass, and by enhancing the oxidation of exogenous fatty acids.

## Supplementary Information

**Additional file 1: Table S1.** Study subject characteristics on biopsy day. **Figure S1.** Cell viability. Results are expressed as the percentage of cell viability relative to the control (= non-treated cells). a) Individual groups represent viability of cells exposed for 96 h to different concentrations of noradrenaline. Data are presented a s the mean ± SEM (*n* = 4 subjects). Values for each experimental condition were measured in triplicates in each subject. b) Individual groups represent viability of cells exposed for 96 h to ethanol (= propofol vehicle; 0.1%), 0.1 mM noradrenaline alone and c) different concentrations (μg/mL) of either propofol alone or mixture of propofol and 0.1 mM noradrenaline. Data are presented as the mean ± SEM (*n* = 7 subjects). Values for each experimental condition were measured in triplicates in each subject. Note: NA = noradrenaline. *** *p* < 0.001 vs. control group. **Figure S2**. Kinetic graph on XF24 Analyzer demonstrates changes after propofol at various concentrations. Real-time measurement of OCR at baseline and after sequential injection of oligomycin, FCCP and Antimycin A. Each data-point represents the mean of 7 independent samples (subjects) measured in tri- or tetraplicates normalized to protein content. Error bars indicate standard error of the mean. Different colours represent different groups exposed to propofol (0; 2.5; 10 µg/mL). **Figure S3.** A) Global mitochondrial parameters. Basal respiration, maximal respiratory capacity, ATP production and non-mitochondrial respiration. N = 7 replicates with 21–28 wells for each condition normalized to protein content. Error bars indicate standard error of the mean. B) Mitochondrial mass calculated as a fraction (%) of a cell surface area in 2D cross-sectional images. Figure S4. Uptake of CO_2_ production and lactic acid metabolism. A) Uptake of [^14^C]palmitic acid. B) [^14^C]lactic acid oxidation. **C)** [^14^C]lactic acid uptake. Note: NA = noradrenaline. Error bars in each graph indicate standard error of the mean. Note: NA = noradrenaline. **Figure S5.** Flow cytometry. Histogram showing MTG intensity of individual cell groups. Data are presented as the mean ± SEM (*n* = 2–3 experiments per each group). Note: NA = noradrenaline. **Figure S6.** Mitochondrial membrane potential. A) Myoblasts after staining with MitoTracker™ Green FM (left), TMRE (in the middle) and after staining of both agents (right). Experiments were performed at least at 60 cells per each group from *n* = 3 independent experiments (cells from 3 individual subjects). B) Determination of ∆ψ_m_ was expressed as TMRE/MTG ratio. The mitochondrial uncoupling agent FCCP was used as a positive control. Note: MTG = MitoTracker™ Green FM; TRME = tetramethylrhodamine ethyl ester; FCCP = carbonyl cyanide-4-(trifluoromethoxy)phenylhydrazone, NA = noradrenaline. Error bars indicate standard error of the mean. *p < 0.05, ***p < 0.001 vs. control group. **Figure S7. **Analysis of lipid droplets. A) Quantification of LD mass by cross-sectional area of BODIPY 493/503 normalized to cell area. B) LD size assessed by cross-sectional area of individual LDs. C) LD number normalized to cell area. Experiments were performed at least at 38 cells per each condition from 2 independent measurements (= 2 individual subjects). Error bars indicate standard error of the mean. ***p < 0.001 vs. control group.

## Data Availability

The datasets used and/or analysed during the current study are available from the corresponding author on reasonable request.
