# Supplementary material for: Effect of noradrenaline on propofol-induced mitochondrial dysfunction in human skeletal muscle cells
Source: Intensive Care Med Exp. 2022 Nov 8;10:47. doi: 10.1186/s40635-022-00474-3 (PMC9643307; doi:10.1186/s40635-022-00474-3)
Supplement: Supplementary file 1 — Additional file 1: Table S1. Study subject characteristics on biopsy day. Figure S1. Cell viability. Results are expressed as the percentage of cell viability relative to the control (= non-treated cells). a) Individual groups represent viability of cells exposed for 96 h to different concentrations of noradrenaline. Data are presented a s the mean ± SEM (n = 4 subjects). Values for each experimental condition were measured in triplicates in each subject. b) Individual groups represent viability of cells exposed for 96 h to ethanol (= propofol vehicle; 0.1%), 0.1 mM noradrenaline alone and c) different concentrations (μg/mL) of either propofol alone or mixture of propofol and 0.1 mM noradrenaline. Data are presented as the mean ± SEM (n = 7 subjects). Values for each experimental condition were measured in triplicates in each subject. Note: NA = noradrenaline. *** p < 0.001 vs. control group. Figure S2. Kinetic graph on XF24 Analyzer demonstrates changes after propofol at various concentrations. Real-time measurement of OCR at baseline and after sequential injection of oligomycin, FCCP and Antimycin A. Each data-point represents the mean of 7 independent samples (subjects) measured in tri- or tetraplicates normalized to protein content. Error bars indicate standard error of the mean. Different colours represent different groups exposed to propofol (0; 2.5; 10 µg/mL). Figure S3. A) Global mitochondrial parameters. Basal respiration, maximal respiratory capacity, ATP production and non-mitochondrial respiration. N = 7 replicates with 21–28 wells for each condition normalized to protein content. Error bars indicate standard error of the mean. B) Mitochondrial mass calculated as a fraction (%) of a cell surface area in 2D cross-sectional images. Figure S4. Uptake of CO2 production and lactic acid metabolism. A) Uptake of [14C]palmitic acid. B) [14C]lactic acid oxidation. C) [14C]lactic acid uptake. Note: NA = noradrenaline. Error bars in each graph indicate standard err [file 40635_2022_474_MOESM1_ESM.docx]

**Additional File 1**

To paper: Krajcova et al. Effect of noradrenaline on propofol-induced mitochondrial dysfunction in human skeletal muscle cells

**Content**

1. List of abbreviations
2. Study subject characteristics (**Table S1**)
3. Details of methods
   1. Isolation and cultivation of human skeletal muscle cells
   2. Cell viability assay
   3. Extracellular flux analysis
   4. Spectrophotometric analysis
   5. Flow cytometry
   6. Cellular staining and microscopic analysis
4. Results
   1. Impact of propofol and noradrenaline on cell viability (**Figure S1**)
   2. Effect of concentration range of propofol on mitochondria (**Figure S2**)
   3. Effect of concentration range of noradrenaline on mitochondria (**Figure S3**)
   4. Palmitate uptake and lactic metabolism (**Figure S4**)
   5. Flow cytometry (**Figure S5)**
   6. Mitochondrial membrane potential (**Figure S6**)
   7. Lipid droplets analysis (**Figure S7**)
5. References
6. **List of abbreviations**

AA = Antimycin A

ASM = acid soluble metabolite

ATP = Adenosine triphosphate

CS = Citrate synthase

DMEM = Dulbecco’s Modified Medium

ETC = Electron transport chain capacity

ETH = Ethanol

FAO = Fatty acid oxidation

FCCP = Carbonyl cyanide-4- (trifluoromethoxy)phenylhydrazone

LD = lipid droplet

OCR = Oxygen consumption rate

MTG = MitoTracker^TM^ Green FM

NA = noradrenaline (norepinephrine)

PA = palmitic acid

PBS = phosphate-buffered saline

PRIS = Propofol infusion syndrome

TMRE = tetramethylrhodamine ethyl ester

1. **Study subject characteristics**

| **Patient** | **Sex** | **Age** | **Weight** | **Height** | **BMI** | **Reason for surgery** | **Comorbidities** | **Biopsy technique** |
| --- | --- | --- | --- | --- | --- | --- | --- | --- |
| 1 | Male | 66 | 93 | 182 | 28.08 | Osteoarthritis of the hip | Arterial hypertension | Open surgery |
| 2 | Female | 74 | 55 | 165 | 20.2 | Femoral neck fracture | Arterial hypertension  Ischemic chronic heart disease  Chronic obstructive pulmonary disease | Open surgery |
| 3 | Female | 50 | 68 | 162 | 25.91 | Congenital hip dysplasia | - | Open surgery |
| 4 | Male | 63 | 95 | 185 | 27.76 | Neck of femur fracture | - | Open surgery |
| 5 | Female | 55 | 68 | 161 | 26.23 | Osteoarthritis of the hip | - | Open surgery |
| 6 | Female | 76 | 76 | 162 | 28.96 | Osteoarthritis of the hip | Arterial hypertension | Open surgery |
| 7 | Female | 76 | 87 | 166 | 31.57 | Osteoarthritis of the hip | Arterial hypertension | Open surgery |
| 8 | Female | 67 | 90 | 172 | 30.42 | Osteoarthritis of the hip | Hyperuricaemia  Hyperlipidemia  Hypothyreosis | Open surgery |
| 9 | Female | 78 | 85 | 163 | 31.99 | Osteoarthritis of the hip | Arterial hypertension  Ovarian cancer | Open surgery |
| 10 | Female | 66 | 84 | 160 | 32.81 | Osteoarthritis of the hip | - | Open surgery |
| 11 | Female | 74 | 82 | 175 | 26.78 | Osteoarthritis of the hip | Arterial hypertension  Hyperlipidemia  Adrenal adenoma  Bronchial asthma | Open surgery |
| 12 | Female | 51 | 84 | 170 | 29.07 | Osteoarthritis of the hip | - | Open surgery |
| 13 | Male | 74 | 70 | 175 | 22.86 | Osteoarthritis of the hip | Hyperuricemia | Open surgery |
| 14 | Female | 70 | 62 | 160 | 24.22 | Neck of femur fracture | Arterial hypertension  Liver cirrhosis | Open surgery |
| 15 | Male | 24 | 78 | 184 | 23.04 | - | - | Bergström needle |
| 16 | Male | 28 | 90 | 186 | 26.015 | - | - | Bergström needle |
| 17 | Male | 24 | 70,8 | 189,5 | 19.72 | - | - | Bergström needle |

**Table S1. Study subject characteristics on biopsy day.**

1. **Details of methods**
   1. **Isolation and cultivation of human skeletal muscle cells**

Skeletal muscle cells were isolated as previously described^1^. Briefly, muscle tissue fragments were initially minced with scissors and digested with 0.25% Trypsin/ 0.68% Collagenase solution for 30 min at 37°C. Fetal bovine serum was then added to stop muscle dissociation and cells were pre-plated on non-coated Petri dishes for 60 minutes to adhere fibroblasts. After that, cells were cultured in “Growth Medium” consisting of Dulbecco’s Modified Medium (DMEM) containing 15 % fetal bovine serum, 5.56 mM glucose, 1 mM pyruvate, 1% solution of 100 U/mL penicillin and 100 µg/mL streptomycin, 0.05 µg/mL fungizone, 0.4 µg/mL dexamethasone, 0.5 mM L-glutamine, 10 µg/mL insulin, 10 ng/mL epidermal and 1 ng/mL fibroblast growth factors at 37°C in a humidified atmosphere of 5% CO2. Upon 80 – 90 % confluency, medium was changed to DMEM with reduced concentration of serum to induce differentiation of myoblasts. After 7 days (or 3 days in substrate oxidation assays) of incubation in this “Differentiation medium” supplemented with 2% Horse serum, 1% solution of penicillin and streptomycin, 25 mM Glucose and 10 μg/mL of insulin, cells were differentiated and formed into multinucleated cylindrical myotubes.

- 1. **Cell viability assay**

***Pre-treatment of cells.*** Upon 80 – 90 % confluency, myoblasts were trypsinized and seeded overnight on gelatine-coated 96-well microplates at the density of 1 x 10^4^ cells/per well for cell viability assay. After 24 hours of incubation in “Growth Medium”, the cells reached confluence and medium was changed to “Differentiation Medium”. Following 7 days of differentiation, myotubes were initially treated for 96 hours with different concentrations of noradrenaline (0.001 - 0.1 mM) to test its impact on the cell viability (see **Figure S1, part A**). Myotubes were then incubated for 96 hours in DMEM containing different concentrations of propofol (1-50 µg/mL) and noradrenaline (0.1 mM; see **Figure S1, part C**). Additional groups of cells were exposed to corresponding concentrations of ethanol vehicle and noradrenaline (0.1 mM; see **Figure S1, part B**). Control cells had no treatment.

***Drugs preparation.*** Propofol stock (2,6-diisopropylphenol; Merck Millipore, Darmstadt, Germany) was prepared fresh before every experiment and diluted in ethanol immediately prior to addition to cells. Noradrenaline tartrate was obtained from Zentiva group a.s., Czech Republic and stored at + 4°C.

***Cell viability assay.*** Cell viability was then analysed using CellTiter 96® AQueous Non-Radioactive Cell Proliferation Assay (Promega, Corp., Madison, WI, USA) according to manufacturer’s instructions^2^. The differentiated myotubes were exposed to various experimental conditions for 96 hours. At the end of incubation period, medium with tested compounds was washed with Phosphate Buffered Saline (PBS) and replaced by medium containing MTS (3-(4,5-dimethylthiazol-2-yl)-5-(3-carboxymethoxyphenyl)-2-(4-sulfophenyl)-2H-tetrazolium). After 2 hours of incubation, the absorbance was recorded spectrophotometrically at 490 nm using spectrophotometer Tecan Infinite M200PRO microplate reader. Cell viability was then calculated as the percentage of non-treated cells as follows: (A _treated cells_ – A _blank_) / (A _non-treated cells_ – A _blank_ ) x 100 %, where A = absorbance. Given that high propofol concentrations impair cell viability, we used only 10 µg/mL for further experiments (see **Figure S1, part C**). Noradrenaline was used at the concentration 0.1 mM, which had no significant impact on cell survival (see **Figure S1, part B**).

- 1. **Extracellular Flux Analysis**

***Pre-treatment of cells.*** Prior to experiment, myoblasts were seeded on gelatine-coated 24-well XF24 V7 cell culture plates at the density of 3 x 10^4^ cells/per well (Agilent Technologies Inc., Santa Clara, CA). After 24 hours, the cells reached confluence and medium was switched to DMEM with 2 % Horse serum to initiate differentiation of cells. After 7 days, differentiated myotubes were exposed to propofol (10 µg/mL), noradrenaline (0.1 mM) or mixture of both agents. All groups were compared to control cells incubated in medium with no drug additions. Each experimental condition was performed in triplicates or tetraplicates. We used ethanol (0.1 %) as a propofol vehicle. We did not observe its effect in this study as we had previously found no impact on mitochondrial metabolism in human skeletal muscle cells at this concentration^9^.

***Measurement of global mitochondrial parameters.*** *Preparation of cells prior to experiment.* On the day of experiment, the instrument was calibrated according to manufacturer’s recommendations^17^. On 24-well XF24 plate, medium with tested compound was removed from each well, cells were washed and maintained in XF Assay Medium (supplemented with 1 mM pyruvate and 4 mM Glucose) for 50-60 minutes in incubator with atmospheric CO_2_ at 37 °C. After that, the cell plate was loaded, and measurement was initiated. *Calculation of global mitochondrial parameters.* Firstly, basal respiration was measured. After that, oligomycin (1 µM) was added to block ATP synthase.  Carbonyl cyanide-4- (trifluoromethoxy)phenylhydrazone (FCCP; 1 µM) was then injected to uncouple cells and determine maximal respiratory capacity. Finally, complex III – inhibitor Antimycin A (AA; 4 µM) was added to block mitochondrial respiration and determine non-mitochondrial respiration. Consequently, after extracting non-mitochondrial respiration we determined basal OCR. ATP production was calculated as a difference between basal respiration and OCR after oligomycin injection. Electron transport chain capacity (ETC) was calculated as OCR after the addition of FCCP minus non-mitochondrial respiration.

***Measurement of fatty acid oxidation.*** *Preparation of cells prior to experiment.* KHB medium (containing 111 mM NaCl, 4.7 mM KCl, 2 mM MgSO4, 1.2 mM Na2HPO4 and distilled water) was prepared according to manufacturer recommendations191. Subsequently, 2.5 mM Glucose and 0.5 mM carnitine were prepared fresh and added to KHB medium. Cells were washed supplemented with KHB medium and subsequently incubated in non-CO2 incubator for 50-60 minutes. *Preparation of sodium palmitate.* Sodium palmitate was used as a substrate for fatty acid utilization and prepared fresh as previously described^3^.

1. **Spectrophotometric analysis**

***Citrate synthase activity.*** Firstly, cell pellets left over from bioenergetic assays were kept frozen for later analysis of citrate synthase (CS), which is an enzyme of tricarboxylic acid cycle catalysing the reaction between acetyl-CoA and oxaloacetate to form citric acid. CS is generally considered a marker of mitochondrial matrix content. CS activity was measured spectrophotometrically using CS Assay kit (Sigma-Aldrich, Corp., St. Louis, MO, USA)^4^.

1. **Flow cytometry**

***Staining of cells.*** After 96-hours treatment, cells were firstly trypsinized, centrifuged at 300 xg and resuspended in 500 µL of pre-warmed PBS containing 200 nM MitoTracker™ Green FM and incubated for 20 minutes at 37°C in the dark. An additional wash with PBS was performed after incubation. Cells were then resuspended in PBS and analysed on BD FACSVerse flow cytometer (BD Biosciences, San Jose, CA, USA; see **Figure S5**). Data were analysed in FlowJo^TM^ v10.8 Software (Becton, Dickinson & Company).

1. **Cellular staining and microscopic analysis**

***Seeding and pre-treatment of cells.*** The cells were seeded, grown and imaged on high-quality microscopy dishes with glass coverslip bottom (μ-Dish 35mm, high Glass Bottom: O35mm, high wall, 2ml, 1.5H; Ibidi). After 4 days of incubation in experimental conditions, confluency was checked and only dishes upon ~ 70 % of confluency were used for microscopy allowing to distinguish individual cells.

***Cellular staining for a live-cell imaging.*** All fluorescent dyes and imaging solution media were purchased from Life Technologies (Gaithersburg, MD), unless otherwise stated.

*Mitochondrial morphology and mass.* The cells were firstly stained with 200 nM green-fluorescent dye MitoTracker™ Green FM, which reflects mitochondrial mass binding to mitochondrial proteins, regardless of its membrane potential^24,25^. During 30 minutes of incubation, cells were kept in 5 % CO2 and 37°C. Myoblasts were then washed with pre-warmed PBS and stained with 5 µg/mL CellMask™ Deep Red Plasma Membrane Stain to label their cellular plasma membrane. 1-2 drops of NucBlue/10 mL of Cell Culture Medium were used to label cell nuclei. After at least 20 minutes of incubation in 5 % CO2 and 37°C, confocal laser scanning microscopy was performed.

*Mitochondrial membrane potential.* Firstly, cells were also stained with 100 nM MitoTracker^TM^ Green FM for 30-40 minutes in 5 % CO2 and 37°C to normalize the values to the mitochondrial volume. Cells were twice washed with pre-warmed PBS. Subsequently, myoblasts were stained with a cell permeant positively-charged red dye tetramethylrhodamine ethyl ester (TMRE; 7 nm), which readily accumulates in the active mitochondria due to their relative negative charge. Cell nuclei were stained with NucBlue dye (1-2 drops/10 mL of Cell Culture Medium). After at least 40 minutes of incubation in 5 % CO2 and 37°C, confocal laser scanning microscopy was performed (see **Figure S6**).

*ROS production.* Cells were loaded with 100 nM MitoTracker Red CM-H2XRos (100 nM) to detect accumulation of mitochondrial specific-reactive oxygen species. After 30 minutes of incubation in 5 % CO2 and 37°C, cells were twice washed with pre-warmed PBS and incubated for at least 40 minutes with Cell Culture Medium containing NucBlue (1-2 drops/10 mL of Cell Culture Medium). After that, confocal laser scanning microscopy was performed.

*Co-localization of mitochondria with lysosomes.* Myoblasts were firstly loaded with 100 nM MitoTracker^TM^ Green FM for 45 minutes in 5 % CO2 and 37°C. Subsequently, the cells were washed with a live-cell imaging solution and stained with 75 nM LysoTracker™ Deep Red for 1 hour in 5 % CO2 and 37°C. Finally, the cells were washed and incubated in a live-cell imaging solution with a nuclear dye NucBlue (1-2 drops/10 mL of live-cell imaging solution).

*Staining of lipid droplets.* Myoblasts were firstly stained with 2 µM BODIPY 493/503 for a 1 hour in 5 % CO2 and 37°C. Cells were then washed with pre-warmed PBS. Subsequently, myoblasts were stained 5 µg/mL CellMask™ Deep Red Plasma Membrane Stain to label their cellular plasma membrane. 1-2 drops of NucBlue/10 mL of Cell Culture Medium were used to label cell nuclei. After at least 20 minutes of incubation in 5 % CO2 and 37°C, confocal laser scanning microscopy was performed. After staining with BODIPY 493/503 (excitation at 488 nm) and CellMask™ Deep Red Plasma Membrane Stain (excitation at 650 nm; see above) confocal laser scanning microscopy was performed (see **Figure S7**). Cellular surface was defined in 2D sections. LD size, number and quantification of LD mass were analysed using 2D cross-sectional area and normalized to cell area in ImageJ (Fiji).

**4. Results**

**a. Impact of propofol and noradrenaline on cell viability**

No significant difference in cell viability was observed between different noradrenaline concentrations (see **Figure S1, part A**). Similarly, propofol-vehicle had no effect on cell viability (see **Figure S1, part B**). Propofol significantly decreased cell survival at greater concentrations (˃ 10 µg/mL). Co-incubation with noradrenaline at the highest concentration (0.1 mM) had no significant effect on propofol-treated cells (see **Figure S1, part C**).


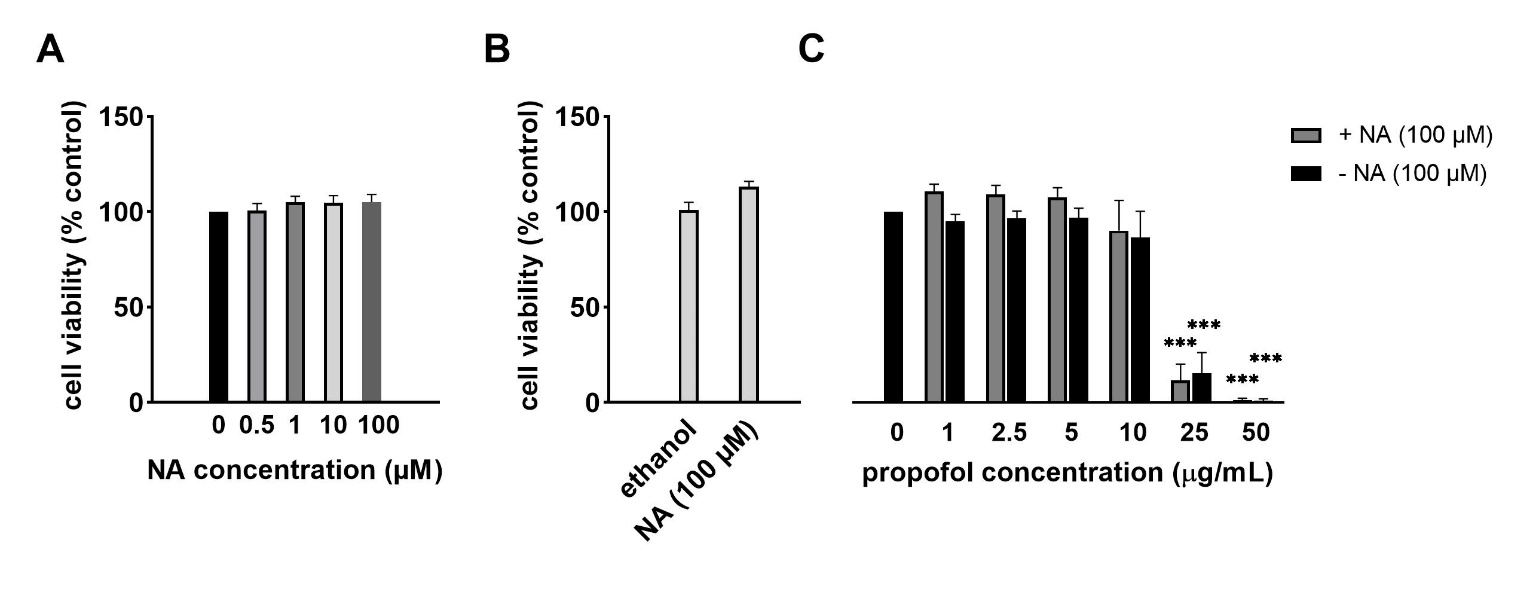


**Figure S1. Cell viability.** Results are expressed as the percentage of cell viability relative to the control (= non-treated cells). **a)** Individual groups represent viability of cells exposed for 96 hours to different concentrations of noradrenaline. Data are presented a s the mean ± SEM (n = 4 subjects). Values for each experimental condition were measured in triplicates in each subject. **b)** Individual groups represent viability of cells exposed for 96 hours to ethanol (= propofol vehicle; 0.1 %), 0.1 mM noradrenaline alone and **c)** different concentrations (μg/mL) of either propofol alone or mixture of propofol and 0.1 mM noradrenaline. Data are presented as the mean ± SEM (n = 7 subjects). Values for each experimental condition were measured in triplicates in each subject. Note: NA = noradrenaline. *** p < 0.001 vs. control group.

1.
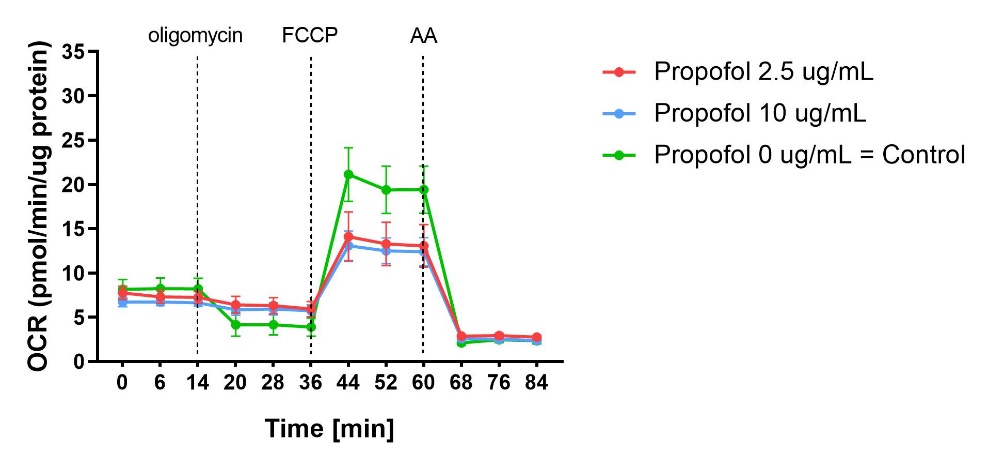
**Effect of concentration range of propofol on mitochondria**

**Figure S2. Kinetic graph on XF24 Analyzer demonstrates changes after propofol at various concentrations.** Real-time measurement of OCR at baseline and after sequential injection of oligomycin, FCCP and Antimycin A. Each data-point represents the mean of 7 independent samples (subjects) measured in tri- or tetraplicates normalized to protein content. Error bars indicate standard error of the mean. Different colours represent different groups exposed to propofol (0; 2.5; 10 µg/mL).

1. **Effect of concentration range of noradrenaline on mitochondria**


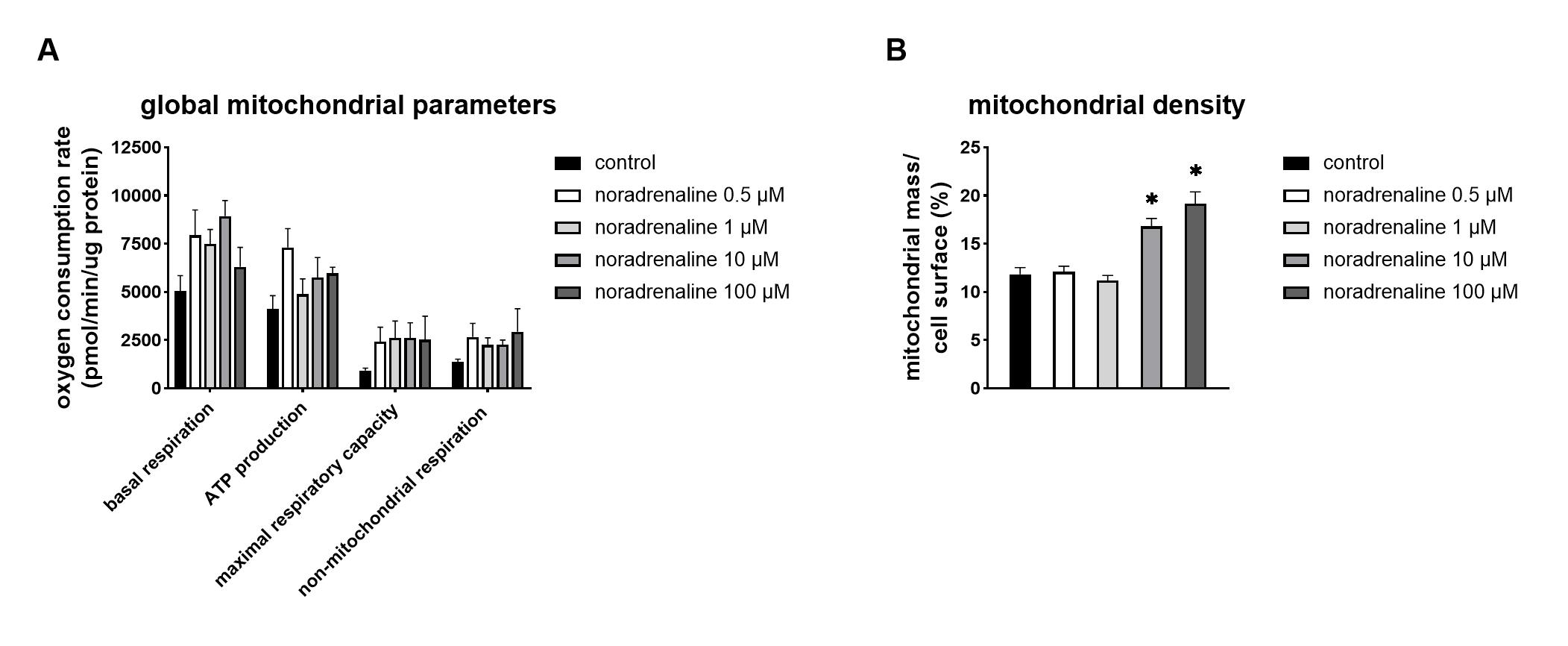


**Figure S3. A) Global mitochondrial parameters.** Basal respiration, maximal respiratory capacity, ATP production and non-mitochondrial respiration. N = 7 replicates with 21-28 wells for each condition normalized to protein content. Error bars indicate standard error of the mean. **B) Mitochondrial mass calculated as a fraction (%) of a cell surface area in 2D cross-sectional images.**

1.
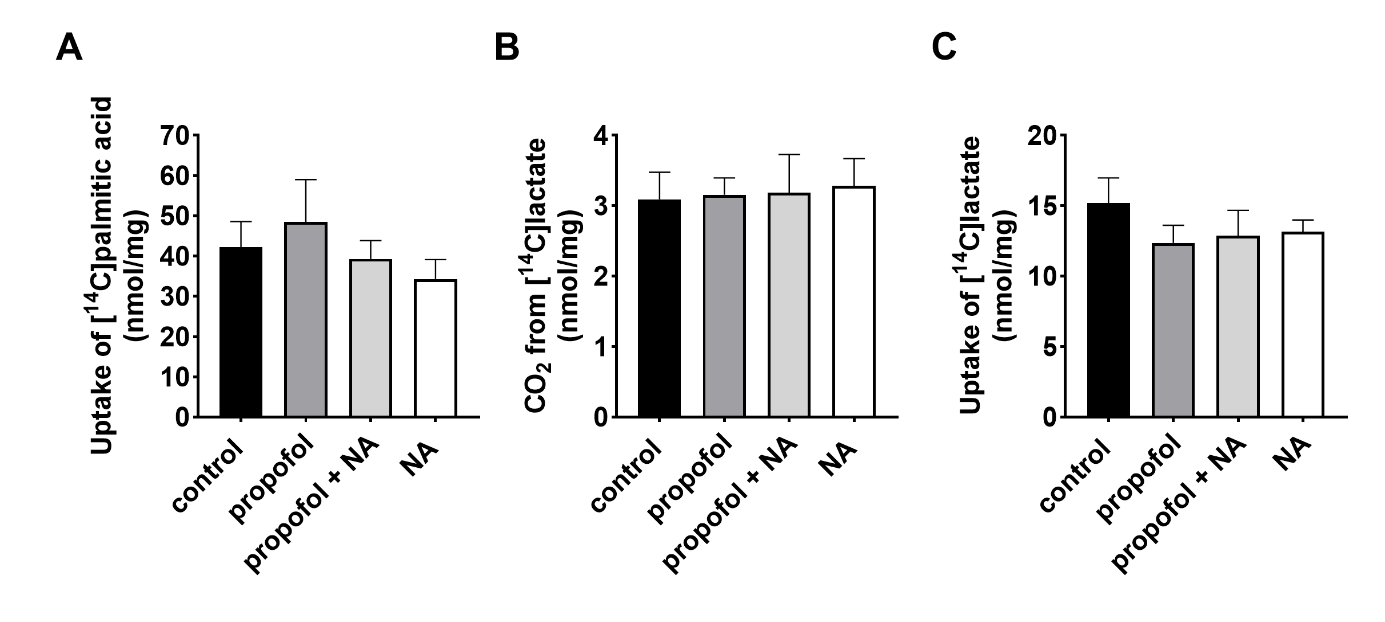
**Palmitate uptake and lactic acid metabolism**

**Figure S4. Uptake of CO_2_ production and lactic acid metabolism.** **A)** Uptake of [^14^C]palmitic acid. **B)** [^14^C]lactic acid oxidation. **C)** [^14^C]lactic acid uptake. Note: NA = noradrenaline. Error bars in each graph indicate standard error of the mean. Note: NA = noradrenaline.

1. **Flow cytometry**


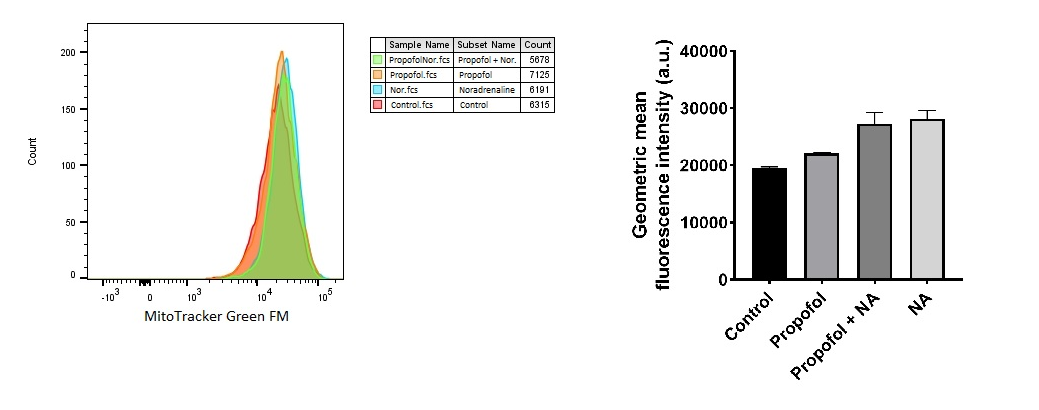


**Figure S5. Flow cytometry.** Histogram showing MTG intensity of individual cell groups. Data are presented as the mean ± SEM (n = 2-3 experiments per each group). Note: NA = noradrenaline.

1. **Mitochondrial membrane potential**


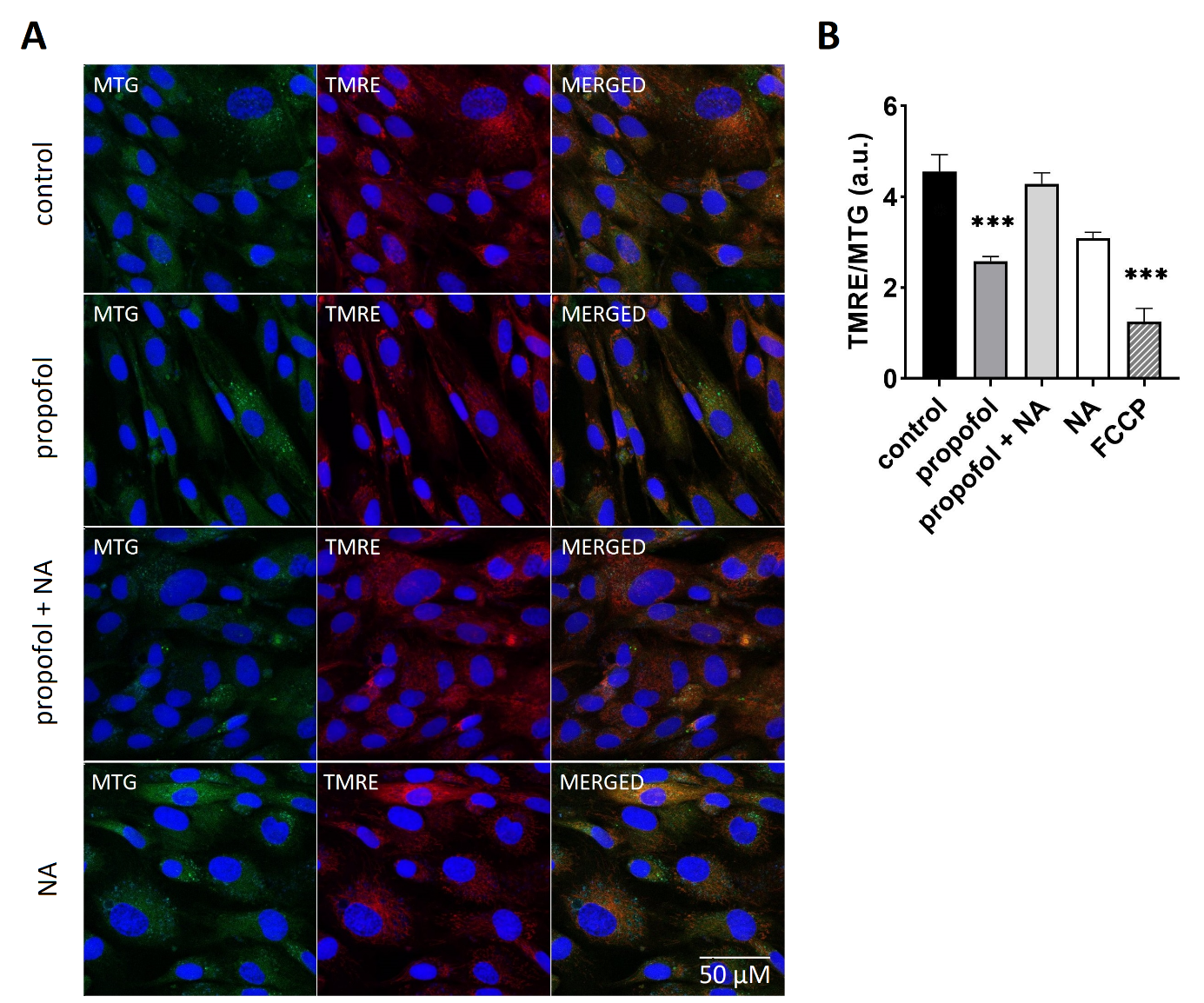


**Figure S6. Mitochondrial membrane potential. A)** Myoblasts after staining with MitoTracker^TM^ Green FM (left), TMRE (in the middle) and after staining of both agents (right). Experiments were performed at least at 60 cells per each group from n = 3 independent experiments (cells from 3 individual subjects). **B)** Determination of Δψ_m_ was expressed as TMRE/MTG ratio. The mitochondrial uncoupling agent FCCP was used as a positive control. Note: MTG = MitoTracker^TM^ Green FM; TRME = tetramethylrhodamine ethyl ester; FCCP = carbonyl cyanide-4-(trifluoromethoxy)phenylhydrazone, NA = noradrenaline. Error bars indicate standard error of the mean. *p < 0.05, *** p < 0.001 vs. control group.

1. **Lipid droplets analysis**

As shown in **Figure S7**, noradrenaline significantly increased LD number and LD area in human myoblasts excluding its stimulatory effect on lipolysis.


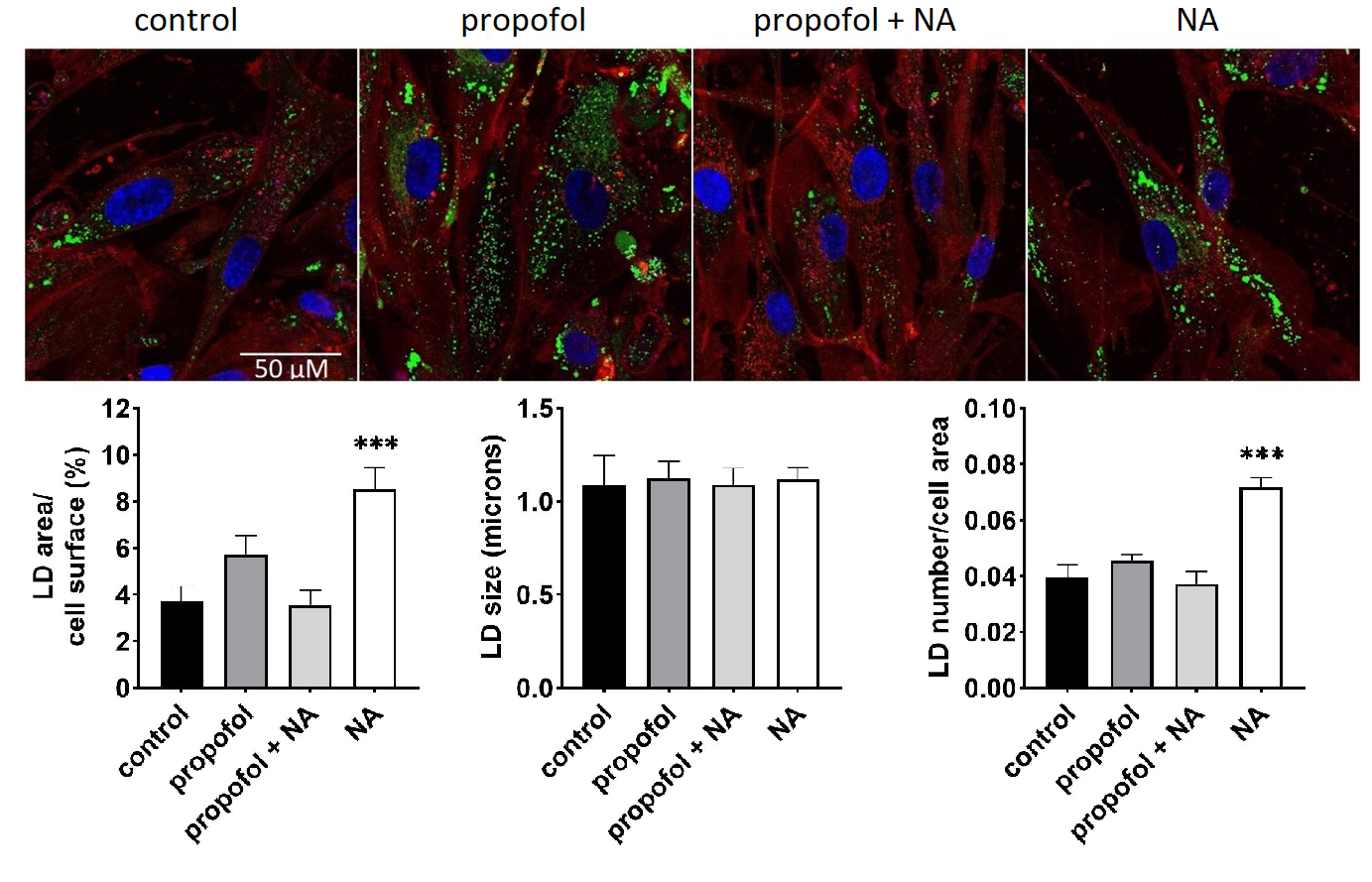


**Figure S7. Analysis of lipid droplets. A)** Quantification of LD mass by cross-sectional area of BODIPY 493/503 normalized to cell area. **B)** LD size assessed by cross-sectional area of individual LDs. **C)** LD number normalized to cell area. Experiments were performed at least at 38 cells per each condition from 2 independent measurements (= 2 individual subjects). Error bars indicate standard error of the mean. *** p < 0.001 vs. control group.

**5. References**

1. Krajcova A, Ziak J, Jiroutkova K, et al. Normalizing glutamine concentration causes mitochondrial uncoupling in an in vitro model of human skeletal muscle. *JPEN J Parenter Enteral Nutr*. 2015;39(2):180–189.

2. Cory AH, Owen TC, Barltrop JA CJ. Use of an aqueous soluble tetrazolium/formazan assay for cell growth assays in culture. *Cancer Commun*. 1991;3(7):207–212.

3. Zhang J, Nuebel E, Wisidagama DRR, et al. Measuring energy metabolism in cultured cells, including human pluripotent stem cells and differentiated cells. *Nat Protoc*. 2012;7(6).

4. Sigma-Aldrich Company: Citrate Synthase Assay Kit. Available at: https://www.sigmaaldrich.com/content/dam/sigma-aldrich/docs/Sigma/Bulletin/cs0720bul.pdf.
